# The Exploration of Therapeutic Antivirals for Human Papillomavirus in the Last 40 Years: Bibliometric Research

**DOI:** 10.3390/pathogens15030265

**Published:** 2026-03-02

**Authors:** Zixiao Jiang, Liangrui Jin, Chengjun Wu, Zhenqing Li, Zhangrong Lou, Peng Qu

**Affiliations:** 1 Faculty of Medicine, Dalian University of Technology, Dalian 116024, China; jiang.zixiao@imbim.uu.se (Z.J.); liangrui.jin@imbim.uu.se (L.J.); 2 School of Health and Life Sciences, Qingdao Central Hospital, University of Health and Rehabilitation Science, Qingdao 266113, China; wcj5532@126.com; 3 Office of Academic Affairs, Chengdu University of Technology, Chengdu 610095, China; zhenqinglee@126.com

**Keywords:** human papillomavirus, antiviral, bibliometrics

## Abstract

Human papillomavirus (HPV) is a well-known carcinogenic DNA virus, responsible for about 4% of all cancer cases globally. Effective antiviral treatments for those who are already infected with HPV are still in their early stages, despite the fact that prophylactic vaccinations have shown impressive success in preventing new infections. Effective treatments for HPV-related malignancies are also hampered by the fact that current articles address a wide spectrum of pathways but lack thorough systematic studies. In this work, we use bibliometric techniques to examine research trends and innovative approaches in the development of HPV antivirals over the last 40 years. Our results are intended to offer insightful information and direct future research into effective antiviral treatments for HPV-induced cancers.

## 1. Introduction

Human papillomavirus (HPV) is an unenveloped DNA virus from the ancient *Papillomaviridae* family, which includes over 200 types [1]. Most HPV infections cause no symptoms while infection with certain high-risk types can persist and cause cancer [2]. HPV is linked to about 4% of all cancer cases worldwide, including cervical cancer, for which over 90% of cases are caused by HPV, leading to about 300,000 deaths each year [3]. In addition, HPV causes penile cancers, head and neck cancers, and other anogenital cancers [4,5]. Collectively, these HPV-associated cancers pose a substantial clinical and economic burden, with costs exceeding USD 700 million [6,7]. Therefore, it is critical to reduce HPV transmission and prevent its cancer-causing effects to improve global public health.

Prophylactic vaccines, which were developed based on HPV’s major capsid protein (L1 protein) [8], have reduced HPV-related diseases in many developed countries by reducing initial HPV infection [9]. In developing regions, however, there was limited vaccination coverage because of high vaccine costs, limited access, and low awareness [10]. As a result, morbidity rates remain high, and more than 600,000 new diagnoses each year cannot benefit from current vaccines. Moreover, current vaccines showed constrained benefits to people that were already infected by HPV [3,11,12]. Thus, there is an urgent need for effective antiviral treatments that can work alongside vaccines and treat established infections.

There have not been approved clinical antiviral therapies that target HPV’s lifecycle/pathological process for HPV-infected patients. However, many experimental attempts aiming to stop HPV-driven cancer have been published [13,14,15]. By understanding how HPV causes cancer, researchers have identified new treatment paths.

E6 and E7 are two well-evidenced oncoproteins produced by high-risk HPV. In the host cells, E6 promotes the degradation of the tumor suppressor protein p53, while E7 inactivates the retinoblastoma protein (pRB). This disruption of key cell cycle checkpoints is a cornerstone of HPV-induced carcinogenesis and caused E6 and E7 to be valuable targets for antiviral drugs [16,17,18]. Moreover, HPV also evades host immune surveillance, for example, the inhibition of innate immune signaling pathways, particularly the interferon (IFN) response and the cytosolic DNA-sensing cGAS-STING pathway. In addition, it has been reported that the replication genes E1 and E2, which initiate HPV genome replication and gene expression, are also considered to be good targets for high-risk HPV therapies because of their roles in the regulation of viral oncogene expression, inhibition of innate immune responses, and interaction with host proliferation-related factors [1,19,20,21].

Based on the mechanisms above, HPV therapeutic antiviral drugs can be mainly categorized as (i) immune response modifiers (e.g., Imiquimod), which activate pattern recognition receptors to counteract viral immune evasion and stimulate a protective immune response; (ii) antiviral agents (e.g., Cidofovir), which aim to inhibit viral replication or restore tumor suppressor function; and (iii) therapeutic vaccines, designed to elicit targeted T-cell responses against the persistently expressed E6 and E7 oncoproteins [22,23,24,25,26,27]. There has been increasing interest in recent decades in developing therapeutics to accelerate the “90-70-90” target (90% girls are vaccinated by the age of 15 years old; 70% women are screened with a high-performance test by the age of 35 and again by 45 years of age; and 90% of women identified with cervical disease received treatment) made by the WHO [28]. However, the existence of thousands of existing publications makes it challenging for researchers to identify overarching trends, prioritize the most promising therapeutic avenues, and avoid duplication of effort. Thus, a systematic analysis of publications is necessary to map the intellectual landscape, identify knowledge gaps, highlight convergent findings, and illuminate emerging innovative strategies, thereby informing a more efficient and targeted direction for researchers.

Bibliometric analysis provides a quantitative and systematic approach for characterizing the large-scale scientific literature. It enables the reconstruction of knowledge structures, the outlining of research evolution, and the identification of research hotspots. These methods allow investigators to extract field-level patterns—such as research hotspots, collaboration networks, and shifts in conceptual focus—from thousands of publications as an addition to typical reviews. Recent methodological developments have further strengthened the reliability of such analyses [29,30]. Optimized citation time window strategies improve the assessment of research impact by balancing short-term citation fluctuations with long-term scholarly influence, thereby reducing distortions caused by temporal citation accumulation. In parallel, scalable techniques for identifying semantically equivalent cue words enhance the detection of conceptual uncertainty and transitions between research topics. Together, these advances improve the precision and interpretive depth of bibliometric evaluations.

Thus, the present study aims to conduct a comprehensive bibliometric analysis of the literature published over the past four decades regarding antiviral strategies against HPV-induced cancers. We seek to integrate scattered findings, identify cutting-edge technologies, and provide a structured foundation to guide future research toward clinically viable antiviral treatments.

## 2. Materials and Methods

### 2.1. Data Collection and Statistics

In the context of the present study, the “antiviral” refers to therapeutic interventions aimed at clearing an established HPV infection, treating HPV-induced lesions, or counteracting the oncogenic activities of viral proteins (notably E6 and E7) in already infected cells. This includes, but is not limited to, antiviral agents (e.g., Cidofovir), immune response modifiers (e.g., Imiquimod), and therapeutic vaccines. Prophylactic measures, such as preventive vaccines, were explicitly excluded from our search strategy and analysis.

The initial dataset was retrieved from the Web of Science Core Collection (WoSCC, https://www.webofscience.com/wos/woscc/) database. The search strategy combined two sets of topic keywords using the Boolean operator AND:

- **Set A** (Therapeutic Antivirals): (“antiviral*” OR “anti-viral” OR “antiviral agent*” OR “antiviral drug*” OR “antiviral therap*” OR “antiviral treatment*” OR cidofovir OR interferon OR ribavirin OR acyclovir OR valacyclovir OR ganciclovir OR imiquimod OR “therapeutic vaccine*” OR “targeted drug*”)
- **Set B** (HPV): (HPV OR “human papillomavirus” OR papillomaviridae OR HPV16 OR HPV18)

The search parameters were set as follows: Publication Years: 1985–2025; Document Type: Article. This query aimed to identify studies focusing on the development of antiviral therapeutics for HPV. The retrieved records were exported in the “Full Record and Cited References” format and saved as plain text files. These files served as the primary dataset for our analysis. After removing duplicates and conducting data cleaning, a total of 2198 records were retained for subsequent analyses.

The software Bibexcel (Version 2016-02-12, https://homepage.univie.ac.at/juan.gorraiz/bibexcel/, Accessed on 12 February 2016) was used to calculate the frequency and co-occurrence of key items (e.g., keywords, countries). Microsoft Excel 2021 (https://www.microsoft.com/, Accessed on 21 August 2024) and OriginLab 2025b (https://www.originlab.com/, Accessed on 18 October 2025) were used for data preprocessing, statistical calculations, and visualizations.

### 2.2. The Construction of Co-Occurrence Networks

All 2198 publication records were imported to CiteSpace (Version.6.3.1, https://citespace.podia.com/, Accessed on 8 October 2025) as a dataset to construct a co-occurrence network among Countries, Institutes, and Categories. The networks were distinguished by using color-coded nodes and edges. Nodes are composed of different colored “tree rings” whose thickness indicates the number of co-occurrences each year. A red ring each year indicates a citation burst. The purple ring is used to indicate the degree of inter-node sexual centrality. A node with high intermediate centrality makes sense because it connects one node to another. The co-occurrence network of Keywords was constructed by VOSviewer (Version 1.6.20, https://www.vosviewer.com, Accessed on 18 September 2021) using a “*.net*” file generated by Bibexcel.

### 2.3. Burst Detection and Cluster Analysis

The processed dataset of 2198 documents were imported into CiteSpace to construct co-occurrence networks for Countries/Regions, Institutions, and Research Categories as what was described by Wu et al. in the year 2022 [31].

In these networks:

- Nodes represented the entities (e.g., Countries, Institutions).
- Edges represented the co-occurrence relationships between entities.
- Node Colors were used to distinguish different clusters.
- Tree Rings within each node indicated the number of publications or co-occurrences each year, with the ring’s thickness proportional to the count.
- A red ring highlighted a year in which the node experienced a citation burst.
- A purple ring indicated that the node had a high betweenness centrality, meaning it played a crucial intermediary role in connecting different parts of the network.

Additionally, VOSviewer (Version 1.6.20, https://www.vosviewer.com/, Accessed on 18 September 2021) was used to construct a Keyword co-occurrence Network. The input file for VOSviewer was a “*.net*” file generated by Bibexcel from the same dataset.

### 2.4. Identification of Core Publications

To identify the most influential publications in the field, the full record dataset was imported into CiteNetExplorer (Version 1.0.0, https://www.citnetexplorer.nl/, Accessed on 20 September 2021) to construct a citation path. Publications were then ranked in descending order based on their internal citation scores within this network. The top 30 most cited publications were selected as the core literature for further in-depth analysis.

## 3. Results and Discussion

After entirely searching from WOSCC database and carefully removing the duplicated or unrelated records, the 2198 publication records (composition of the records was listed in Table 1) were finally be included to further analysis. To construct a general view of this field, we quantify and statist the basic information of the records. The results were shown below.

### 3.1. Quantitative Analysis of Basic Information

#### 3.1.1. Annual Publication Trend

**Figure 1:**
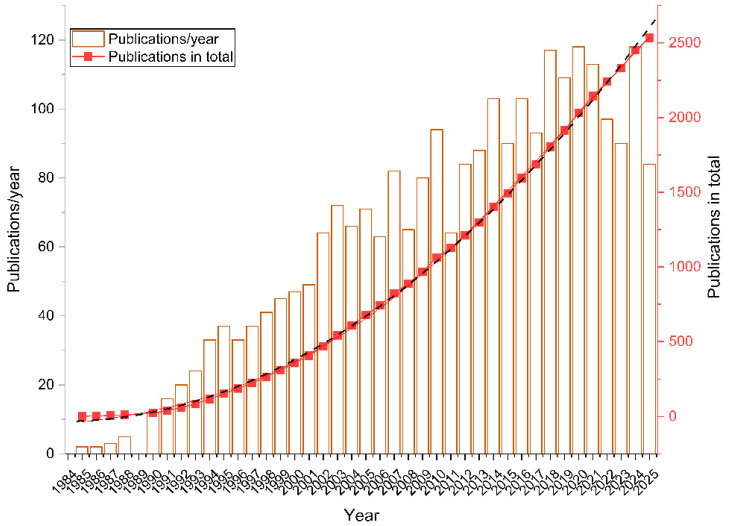
Annual publication trend. The brown bars represented the counts of the annual publications. The red dots represented the accumulation of the publications. The black curve in dash was the fitting curve of cumulative publications.

We quantified annual and cumulative publication counts in the field of therapeutic HPV antiviral development from 1985 to 2025 (Figure 1). The annual number of publications increased from just 2 per year in 1985–1986 to a peak of 117 in 2018, reflecting growing research interest over time. Notable declines were observed in specific years, including 2006 (63 articles), 2008 (65), 2011 (64), 2022 (97), and 2023 (90). Cumulative publication growth followed a strongly fitted polynomial trend (Formula 1).

(1) $$y=1.5261x^{2}+108789x-41.532\;R^{2}=0.9994$$

**Formula 1.** The polynomial function of the article accumulations. y—the number of the accumulated publications at the time point. x—years from 1985.

Annual publication counts serve as an indicator of field activity. A rapid rise from 1985 to 2003 aligns with growing recognition of HPV’s oncogenic potential. However, there are two significant fluctuations in year 2006 and 2019, respectively. fluctuations in publication counts may coincide with major events in the field—such as the introduction of prophylactic HPV vaccines [32,33], or the global shift of virology research during the COVID-19 pandemic [34]—but that these associations do not imply direct causation.

Despite these fluctuations, the cumulative publication curve suggests a consistent upward trajectory, indicative of sustained developmental interest. However, the relatively modest total output highlights persistent challenges—such as methodological or theoretical limitations—that warrant further investigation to accelerate effective antiviral design. Together, these findings underscore a continuing demand for HPV therapeutics, though research priority appears susceptible to competing public health emergencies.

#### 3.1.2. Analysis of Published Journals

To gain insight into the intellectual landscape of anti-HPV therapy research, we identified the top 10 most productive journals in this field (Table 2), which reveals the interdisciplinary nature and evolving focus of HPV antiviral research. *Journal of Virology* leads with 81 publications, establishing virology as the foundational discipline. The significant presence of high-impact (Q1) journals in immunology (*Frontiers in Immunology*, 5-year IF: 6.8) and oncology (*International Journal of Cancer*, 5-year IF: 5.9) underscores the field’s translational ambition and scientific requirement.

The publisher distribution, with 40% of top journals belonging to *ELSEVIER*, indicates consolidated knowledge dissemination in established life sciences domains. However, the prominence of broad-scope journals like *PLOS One* and *Scientific Reports* suggests the field also benefits from wide, cross-disciplinary visibility.

The distribution of journals reveals a clear shift in research priorities. Most core publications appear in immunology and virology journals, reflecting a strong focus on understanding HPV’s immune evasion and developing immune-based therapies such as immunomodulators and therapeutic vaccines. In contrast, pharmacology and drug delivery journals are underrepresented. This suggests a gap—and an opportunity—for future studies to explore how antiviral agents can be better delivered and absorbed, which is essential for improving their clinical effectiveness.

### 3.2. Country, Institution, and Author Cooperation Network

**Figure 2:**
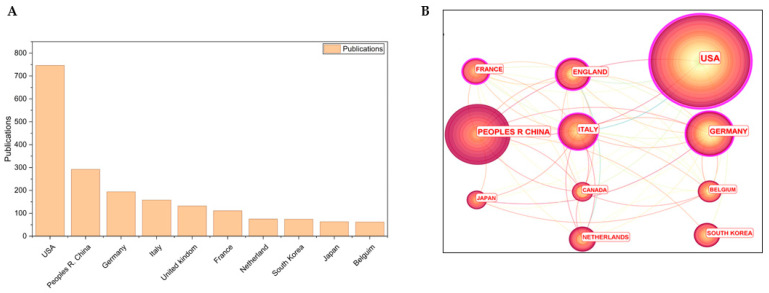
Publication number (**A**) and cooperative relationship among countries (**B**). Node size: publication count. Node/link color: publication year (darker = more recent).

Next, we analyzed international collaboration patterns in HPV therapeutic research (Figure 2A,B). Publication output varied substantially among countries. Seven countries published over 100 articles each over the past 40 years. The United States led with 746 publications, followed by China (292), Germany (194), Italy (158), the United Kingdom (132), and France (111). As illustrated in Figure 2B, collaborative networks are widespread, with stronger linkages observed among Western European countries. Interestingly, although the U.S. and China produced the most publications, they exhibited relatively limited collaboration with other countries. The strongest partnerships have formed predominantly within the last decade.

Publication activity in developed countries appears temporally dispersed, whereas China—a relative latecomer—showed a notable concentration of publications within the past five years. This geographic asymmetry mirrors patterns observed during the development of prophylactic HPV vaccines. Although international collaboration exists, top-producing countries tend to conduct research independently, possibly reflecting divergent national HPV transmission profiles and intervention strategies [35,36]. The widespread adoption of vaccination programs—which have reduced HPV incidence over time [37,38]—may help explain the growing interest in therapeutic options, especially as prophylactic vaccines are ineffective against established infections. China’s distinctive pattern, with a sharp rise in publications coinciding with the introduction of its domestically developed prophylactic vaccine (Cecolin^®^) [39], suggests a strategic shift toward therapeutic research in anticipation of future declines in HPV incidence. This implicates both national public health planning and scientific capacity-building as drivers of research focus.

**Figure 3:**
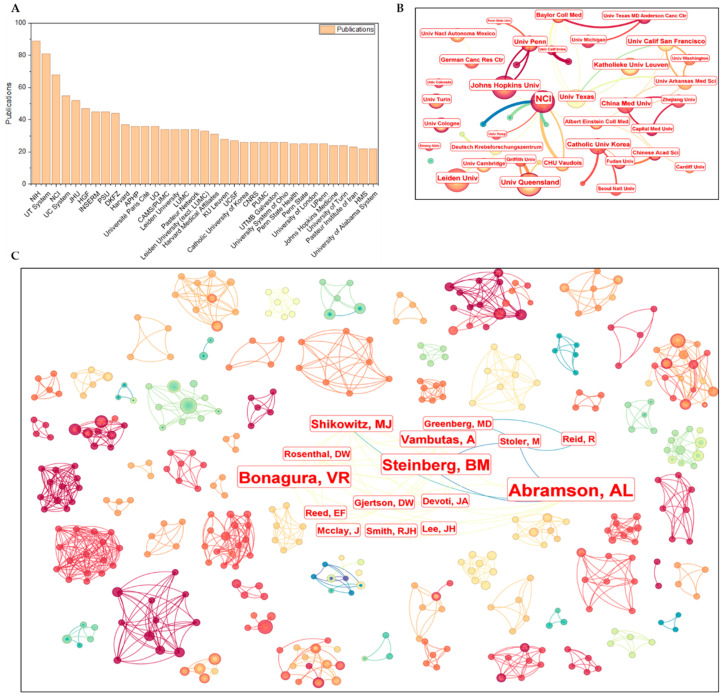
Publication number (**A**), cooperative relationship among institutions (**B**), and authors (**C**) Node size: publication count. Node/link color: publication year (darker = more recent). NIH—National institutes of health, USA; NCI—NIH National cancer institute, USA.

The institutions contributing at least 1% of the total publications are presented in Figure 3A,B. Among the 35 institutions meeting this threshold, 17 were from the United States, including all the top five—*the National Institutes of Health* (NIH), *the University of Texas system*, *the National Cancer Institute* (NCI), *the University of California system*, and *Johns Hopkins University*—which together accounted for 15.7% of the publications. Eleven institutions were based in Europe (18.7% of publications), and four were in Asia (5.0%). Collaborative networks among these institutions appeared stable and closely interconnected (Figure 3B), particularly within national boundaries. Unlike the stable and specialized profiles observed among European and U.S. institutions, those in Asia—such as those from China and South Korea—tended to exhibit broader research interests across multiple institutions, resulting in a more divergent and less converged collaborative structure.

This pattern is consistent with the author-level collaboration network illustrated in Figure 3C, revealing modest yet stable collaborative relationships among researchers worldwide. Two distinct forms of collaboration are evident in the author co-occurrence network: (a) team-based structures centered around principal investigators, characterized by sub-networks with high-centrality nodes, and (b) decentralized collaborations among equal contributors, represented by sub-networks with more random node centrality. As indicated by the color gradient in Figure 3C, most collaborations are clustered within specific time intervals. Specifically, nodes with bluer hues correspond to earlier collaborations, while redder nodes indicate more recent ones. Notably, one network—annotated with author names in Figure 3C—exhibits a sustained collaboration spanning over ten years. To identify prominent investigators, the top 30 most prolific authors based on publication output are summarized in Table 3.

To summary, the decentralized collaboration patterns across *Countries*, *Institutions*, and *Authors* reflect an establishing yet geographically constrained research landscape.

### 3.3. Identification of Core Bibliography

To identify core publications that have contributed significantly to the development of this field, citation records were analyzed using CitNetExplorer (v1.0.0) and ranked by internal citation score (Table 4). The most highly cited publications emphasize that HPV persistence and carcinogenesis are driven by sophisticated immune evasion mechanisms, primarily mediated by the viral oncoproteins E6 and E7. These proteins disrupt interferon signaling by targeting key molecules such as IRF-3 [40], IRF-1 [41], Tyk2 [42], STAT-1 [43,44], and *p*48 [45], and inhibit the cGAS-STING DNA sensing pathway [46]. Moreover, HPV oncoproteins could promote an immunosuppressive microenvironment characterized by Th2 skewing and regulatory T-cell infiltration [47]. Therapeutic advances have emerged through three complementary strategies: (a) immune response modifiers [48] (e.g., imiquimod [22]), which activate innate immunity via TLR7 [49] and promote Th1-dominant cytokine response [50]; (b) antiviral agents such as cidofovir [24], which inhibit viral replication and restore tumor suppressor function; and (c) therapeutic vaccines targeting E6/E7 [27], capable of inducing specific and durable T-cell responses associated with clinical regression. Together, these approaches underscore that successful clearance of HPV infection requires multimodal strategies to counteract viral immune suppression and robustly engage both innate and adaptive immunity.

These core publications (Table 4) reveal a coherent and evolving narrative of the field’s understanding of HPV pathogenesis and therapeutic intervention. The highly cited works collectively underscore that the persistence of HPV and progression to cancer are fundamentally enabled by the virus’s ability to suppress host innate and adaptive immunity, a function masterminded by the E6 and E7 oncoproteins.

Early foundational studies identified the direct targeting of the interferon (IFN) signaling cascade as a primary immune evasion strategy. For instance, seminal papers demonstrated that E6 binds and inhibits interferon regulatory factor-3 (IRF-3), a key transcription factor for IFN production [40], while E7 was shown to abrogate signaling by interferon-α [42,45] and inactivate IRF-1 [41]. Subsequent research expanded this paradigm, revealing that HPV oncoproteins also impair JAK-STAT signaling by associating with Tyk2 [42] and suppressing STAT-1 expression [43,44]. More recently, the focus has shifted to the cGAS-STING DNA-sensing pathway [51], with landmark publications showing that HPV oncogenes antagonize this critical cytosolic surveillance mechanism to avoid detection of viral DNA [46,52].

This mechanistic understanding directly informed the three major therapeutic strategies reflected in the core literature: (i) host Immune Activation using agents like Imiquimod, a TLR7 agonist that induces a Th1-dominant cytokine milieu [22,50,53], effectively “reversing” the local immune suppression; (ii) Antiviral action with drugs like Cidofovir, which not only inhibits viral replication but has been shown to restore p53 function and enhance the sensitivity of HPV-positive cells to other therapies [24,25]; and (iii) Therapeutic Vaccination designed to elicit cytotoxic T-lymphocytes specifically targeting E6 and E7 oncoproteins, with clinical efficacy demonstrated in trials of DNA-based vaccines like VGX-3100 [54,55]. The progression of these strategies from basic discovery to clinical validation, as captured by the citation network, highlights a field that is increasingly translational and mechanism-driven.

It is also important to emphasize that high citation counts reflect the influence of core publications in shaping the field, for example, by proposing immune evasion mechanisms or identifying antiviral targets. However, citation impact does not imply that these targets have been experimentally validated or translated into clinical use. Many of these studies offer conceptual frameworks that have inspired further research. Notably, several highly cited papers link HPV virulence to key cellular pathways such as NF-κB and p53. These pathways are essential for normal cell function, so targeting those pathways requires caution to avoid potential adverse effects.

**Table 4:** The top 30 most cited core publications on HPV antivirals.

NO	Article	Journal	Year	Cite Score
1	Vaccination against HPV-16 oncoproteins for vulvar intraepithelial neoplasia [54]	New England Journal of medicine	2009	89
2	Human papillomavirus 16 E6 oncoprotein binds to interferon regulatory factor-3 and inhibits its transcriptional activity [40]	Genes & development	1998	66
3	Papillomavirus type 16 oncogenes downregulate expression of interferon-responsive genes and upregulate proliferation-associated and NF-κB responsive genes in cervical keratinocytes [56]	Journal of Virology	2001	49
4	Safety, efficacy, and immunogenicity of vgx-3100, a therapeutic synthetic DNA vaccine targeting human papillomavirus 16 and 18 E6 and E7 proteins for cervical intraepithelial neoplasia 2/3: a randomized, double-blind, placebo-controlled phase 2b trial [55]	Lancet	2015	46
5	Treatment of severe laryngeal papillomatosis with intralesional injections of cidofovir [(s)-1-(3-hydroxy-2-phosphonylmethoxypropy) cytosine] [24]	Journal of Medical Virology	1998	45
6	Treatment of genital warts with an immune-response modifier (imiquimod) [22]	Journal of the American Academy of Dermatology	1998	45
7	Microarray analysis identifies interferon-inducible genes and STAT-1 as major transcriptional targets of human papillomavirus type 31 [43]	Journal of Virology	2000	45
8	Treatment of vulvar intraepithelial neoplasia with topical imiquimod [23]	New England Journal of Medicine	2008	45
9	The human papillomavirus E7 oncoprotein abrogates signaling mediated by interferon-α [45]	Virology	1999	44
10	Inactivation of interferon regulatory factor-1 Tumor suppressor protein by HPV E7 oncoprotein implication for the E7-mediated immune evasion mechanism in cervical carcinogenesis [41]	Journal of Biological Chemistry	2000	44
11	A randomized, controlled, molecular study of condylomata acuminata clearance during treatment with imiquimod [53]	Journal of Infectious Diseases	1998	39
12	The human papilloma virus (HPV)-18 E6 oncoprotein physically associates with TYK2 and impairs JAK-STAT activation by interferon-α [42]	Oncogene	1999	39
13	Phase II trial of imiquimod and HPV therapeutic vaccination in patients with vulval intraepithelial neoplasia [57]	British Journal of Cancer	2010	39
14	Cytokine production patterns in cervical intraepithelial neoplasia: association with human papillomavirus infection [50]	Journal of the National Cancer Institute	1997	34
15	Imiquimod, a patient-applied immune response modifier for treatment of external genital warts [58]	Antimicrobial Agents and Chemotherapy	1998	32
16	High-risk human papillomaviruses repress constitutive kappa interferon transcription via E6 to prevent pathogen recognition receptor and antiviral gene expression [59]	Journal of Virology	2011	32
17	Successful treatment of a squamous papilloma of the hypopharynx–esophagus by local injections of (s)-1-(3-hydroxy-2-phosphonylmethoxypropyl) cytosine [60]	Journal of Medical Virology	1995	31
18	Antiproliferative effects of acyclic nucleoside phosphonates on human papillomavirus (HPV)-harboring cell lines compared with HPV-negative cell lines [61]	Oncology Research	1998	28
19	Antiviral agent cidofovir restores p53 function and enhances the radiosensitivity in HPV-associated cancers [25]	Oncogene	2002	28
20	Phase II double-blind, placebo-controlled study of the safety and efficacy of cidofovir topical gel for the treatment of patients with human papillomavirus infection [26]	Clinical Infectious diseases	2001	27
21	Immune responses to human papillomavirus [62]	Vaccine	2006	27
22	Leukoregulin and gamma-interferon inhibit Human papillomavirus type-16 gene transcription in human papillomavirus-immortalized human cervical cells [27]	Cancer Research	1992	26
23	Detection of human papillomavirus (HPV) 16-specific CD4^+^ T-cell immunity in patients with persistent hpv16-induced vulvar intraepithelial neoplasia in relation to clinical impact of imiquimod treatment [47]	Clinical Cancer Research	2005	26
24	Carrageenan is a potent inhibitor of papillomavirus infection [63]	PLoS Patho-gens	2006	26
25	Success or failure of vaccination for hpv16-positive vulvar lesions correlates with kinetics and phenotype of induced T-cell responses [64]	Proceedings of the National Academy of Sciences of the United States of America	2010	26
26	Selective inhibition of human papillomavirus-induced cell proliferation by (s)-1-[3-hydroxy-2-(phosphonyl methoxy)propyl]cytosine [65]	Antimicrobial Agents and Chemotherapy	1999	25
27	HPV: from infection to cancer [49]	Biochemical Society Transactions	2007	25
28	DNA tumor virus oncogenes antagonize the cGAS-STING DNA-sensing pathway [46]	Science	2015	25
29	Suppression of STAT-1 expression by human papillomaviruses is necessary for differentiation-dependent genome amplification and plasmid maintenance [44]	Journal of Virology	2011	24
30	Vaccination against oncoproteins of HPV16 for noninvasive vulvar/vaginal lesions: lesion clearance is related to the strength of the T-cell response [66]	Clinical Cancer Research	2016	24

### 3.4. Tracing the Evolution of the Disciplines

**Figure 4:**
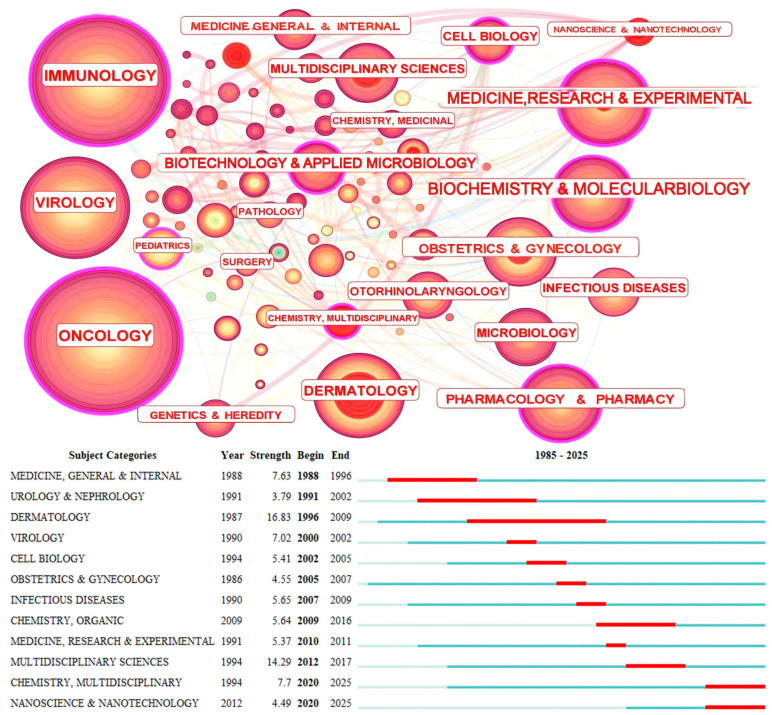
Citation network and burst of category. Node size: publication count. Node/link color: publication year (darker = more recent). Red dots in the middle of the nodes—citation burst.

The evolutionary trajectory and research frontiers in HPV therapy development have been further explored through the construction of citation networks and the detection of burst terms in *Categories* and *Keywords*. As shown in Figure 4, this field has attracted widespread interest across multiple disciplines, with significant contributions from *Cancer biology*, *Immunology*, *Pharmacology*, *Virology*, and *Chemistry*. Among these, 12 subject categories exhibited citation bursts. The earliest bursts occurred in *Medicine (General & Internal)*, *Urology & Nephrology*, and *Dermatology*, lasting between 8 and 13 years. In contrast, the most recent bursts—observed in *Nanotechnology* and *Multidisciplinary Chemistry*—emerged in 2020 and persist to the present. This shift in active research categories reflects an evolution from intradisciplinary approaches to increasingly interdisciplinary strategies. The emergence of nanotechnology may indicate a growing focus on precise therapeutic targeting and efforts toward translational applications. Certain domains, including *Virology*, *Immunology*, and *Oncology*, did not exhibit citation bursts, suggesting sustained and continuous interest throughout the entire period. These patterns imply that key aspects of HPV pathology remain incompletely understood, and that novel therapeutic strategies are likely to continue emerging in the future.

**Figure 5:**
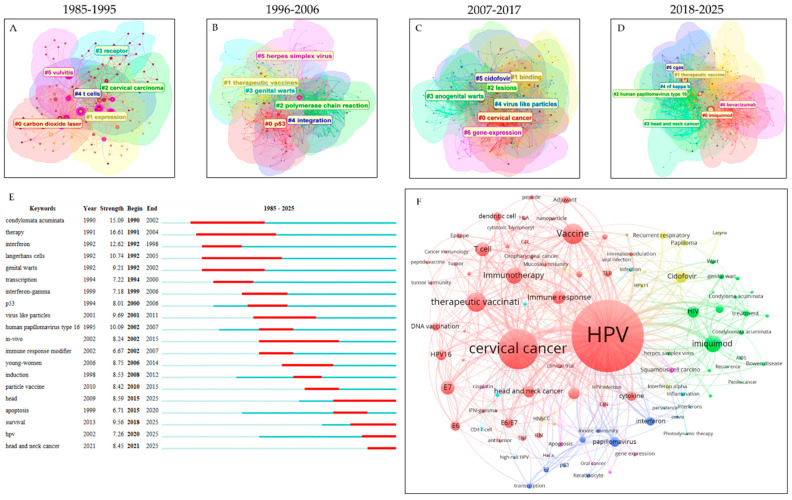
Evolution of key words in the discipline. (**A**–**D**) Clustered keyword co-occurrence networks. Node size: publication count. Node/link color: publication year (darker = more recent). Clusters were distinct by colors. (**E**) Keyword citation burst. (**F**) Co-occurrence networks of keywords used in all publications.

A strong correlation among keywords was observed within the clustered co-occurrence network of the discipline (Figure 5). To trace the temporal evolution of research themes, the network was reconstructed using reference data divided into four time slices and subsequently clustered using CiteSpace (Figure 5A–D). In the first decade, keywords were grouped into five major clusters: #1 *gene expression*, #2 *cervical carcinoma*, #3 *receptors*, #4 *T-cells*, and #5 *vulvitis* (Figure 5A). In the following decade, new research hotspots emerged, including #0 *p53*, #1 *therapeutic vaccines*, #2 *polymerase chain reaction* (PCR), and #3 *genital warts* (Figure 5B). Between 2007 and 2017, more therapy-specific clusters appeared, such as #5 *cidofovir* and #4 *virus-like particles*, alongside persistent themes like #0 *cervical cancer*, #3 *anogenital warts*, and #6 *gene expression* (Figure 5C). The most recent period has been marked by investigations into novel therapeutic targets (e.g., #4 *NF-κB* and #5 *cGAS*) and agents (e.g., #0 *imiquimod* and #6 *bevacizumab*), accompanied by growing attention to #3 *head and neck cancer* (Figure 5D).

To further elucidate evolutionary trends in research focus, the 25 keywords with the strongest citation bursts are displayed in Figure 5E. The evolution of HPV antiviral development exhibits a progressive shift from early emphasis on condylomata acuminata (prevalent during 1990–2002) to recent interest in head and neck cancer (2021–2025). Most bursts persisted for approximately a decade, and overlapping intervals between consecutive terms reflect strong thematic continuity within the field. Consistent with the cluster analysis, the burst detection reveals a trend toward more precise molecular targeting and expanding clinical indications.

Finally, to provide an integrated conceptual overview, a keyword co-occurrence network was constructed using VOSviewer after merging synonymous terms and removing duplicates (Figure 5F). The network reveals one dominant cluster related to vaccine development (red) and three subsidiary clusters associated with specific agents: interferon (blue), cidofovir (yellow), and imiquimod (green). The structure also suggests distinct therapeutic preferences; for example, imiquimod appears frequently in contexts involving co-infections, whereas cidofovir, though typically applied topically, is also investigated for low-risk HPV-associated respiratory papillomatosis.

The keyword evolution and burst analysis collectively illustrate a dynamic and maturing research landscape in HPV therapeutics. The early focus on virological basics and generalized immunology has progressively given way to mechanism-driven drug discovery [67] and targeted therapies [68]. The emergence of themes such as NF-κB [56,69] and cGAS signaling [21,52,70,71,72] indicates a growing emphasis on intracellular innate immune pathways, while the sustained interest in vaccines and virus-like particles underscores the continued importance of immunoprevention [73]. The shift in disease focus from cervical cancer to head and neck cancers reflects both clinical need and expanding therapeutic applicability. The co-occurrence network further highlights the diversity of intervention strategies, from immunomodulators to antiviral agents, each occupying distinct niches within the treatment spectrum. The segregated yet connected clustering pattern suggests that while research subcommunities have specialized, cross-disciplinary awareness remains active. Overall, these findings depict a field that is increasingly interdisciplinary, translational, and biomarker-informed. Future efforts will likely continue to integrate mechanistic insight with clinical innovation, particularly in personalized therapies and combination regimens.

## 4. Conclusions and Perspectives

This bibliometric analysis maps the intellectual landscape and evolving trajectory of antiviral research for HPV-induced cancers over the past four decades. Our findings illustrate a clear paradigm shift from descriptive virology and symptom management towards a deep, mechanism-based understanding of HPV–host interactions, paving the way for targeted therapeutic strategies.

The analysis of publication trends, collaboration networks, and conceptual evolution consistently highlights the centrality of HPV’s immune evasion mechanisms, masterminded by the E6 and E7 oncoproteins. The field’s progression is evident in the keyword and category bursts, moving from general virology and clinical observation to specific molecular targets (e.g., NF-κB, cGAS-STING) and innovative platforms like nanotechnology. The sustained, non-bursting activity in core fields like Immunology and Virology indicates a solid foundation upon which new interdisciplinary approaches are being built.

### 4.1. Future Research Directions

Despite significant progress, challenges remain. Based on the gaps and emerging trends identified in our analysis, we propose the following concrete directions for future research:

#### 4.1.1. Novel Target Discovery

While E6 and E7 remain prime targets, the intricate network of host–virus interactions is not fully elucidated. Future work should employ systematic approaches (e.g., CRISPR screens, proteomics) to identify novel host dependency factors that can be therapeutically exploited.

#### 4.1.2. Advanced Delivery and Formulations

HPV infections are very common and there is not an adequate medical treatment. Despite that several agents (e.g., nucleoside analogs, oncoprotein inhibitors and antineoplastic agent) have currently been used for treatments in HPV-associated lesions both clinically and pre-clinically [74,75,76,77], the therapeutic efficiency and related mechanisms should be further investigated to avoid inaccurate inferences.

The emergence of “Nanotechnology” as a burst term signals a growing focus on overcoming delivery challenges. Thus, Future efforts should prioritize the development of smart nanocarriers to improve the bioavailability, tumor targeting, and intracellular delivery of existing agents (e.g., Cidofovir) and novel nucleic acid-based therapeutics. Virus-like particles (VLPs) have obtained wide interest in HPV therapy development. Although prophylactic HPV VLP vaccines do not directly eliminate established HPV infection and current vaccines showed constrained benefits to people that already infected by HPV [3,11,12], certain studies have reported measurable post-treatment benefits or “primary prophylactic” effects in women treated for HPV-associated lesions [78,79]. In specific clinical windows, VLP-based vaccination has also been explored as an adjunctive therapeutic intervention [80]. Considering the lack of virologic confirmation, further preclinical/clinical investigations are needed to decipher the therapeutic efficiency of the current HPV capsid protein based VLPs.

On the other hand, VLPs are increasingly used as delivery platforms for therapeutic agents, including nucleic acids and immunomodulators [81,82]. This emerging application positions VLPs within the broader therapeutic development pipeline, even though they are not used as antivirals directly. In addition, other newly established platforms provided new opportunities for HPV therapeutic development [83].

Taken together, the accumulated evidence over the past four decades has progressively clarified the conceptual and methodological pathways for HPV antiviral development.

#### 4.1.3. Rational Combination Therapies

Co-occurrence networks reveal distinct clusters for vaccines, immunomodulators, and direct antivirals, suggesting complementary mechanisms. Combining systemic immune activation (e.g., therapeutic vaccines) with local immune modulation (e.g., Imiquimod) may yield synergistic effects and warrants systematic evaluation in future clinical trials.

#### 4.1.4. Expanding Clinical Scope

The strong citation burst for “head and neck cancer” reflects the shifting epidemiology of HPV-associated disease. Oropharyngeal cancers exhibit distinct microenvironmental features compared with anogenital cancers, underscoring the need to adapt antiviral strategies to these emerging clinical contexts.4.1.5 Fostering Global Collaboration.

The observed geographic asymmetry in research output and the relatively limited international collaboration between top-producing countries represent a missed opportunity. Purposeful North–South and cross-continental partnerships, particularly to include regions with high HPV burden, are essential to ensure that research outcomes are globally relevant and translatable.

### 4.2. Limitations of the Study and Perspectives

This bibliometric analysis offers a structured, data-driven overview of four decades of HPV antiviral research. However, several methodological limitations must be clarified and discussed.

First, Bibliometric analysis evaluates the influence of publications mainly through citation-based indicators [29]. Because citations accumulate over time, older studies naturally gain more references and may appear more important within the citation network than newer work [30]. This can lead investigators to identify well-established or classic studies more easily than recent research that may represent state-of-the-art advances but has not yet been widely cited. To reduce this temporal imbalance, we applied citation-burst detection to highlight recent publications that have attracted rapid attention. Although this approach helps identify emerging topics, it cannot fully offset the inherent advantage of long-standing literature. Consequently, the key works identified in Section 3.3 reflect established citation trends but may not fully capture the most recent or innovative studies. This underscores the need for careful, independent evaluation of newly published research beyond what bibliometric indicators alone can provide.

Second, it is critical to separate the scope of this study from that of a typical narrative review. Narrative reviews offer a qualitative summary of mechanisms and clinical findings [84], whereas bibliometric approaches focus on macro-level patterns like collaboration networks, thematic evolution, and publication trends. In this sense, our analysis depicts the larger research environment, whereas narrative reviews delve into individual scientific pathways. Instead of being interchangeable, these strategies are complementary.

Third, the data source limits the bibliometric analysis. We relied solely on the Web of Science Core Collection, which may have excluded important papers published in non-indexed journals, non-English languages, preprints, or clinical trial databases. Furthermore, search string constraints may have unintentionally left out certain relevant papers despite careful query design.

Lastly, metrics based on citations show scholarly interest rather than necessarily scientific significance or translational usefulness. In other words, highly cited publications should not be seen as necessarily excellent. Unlike traditional reviews that focus on the detailed content and interpretation of individual studies, bibliometric methods—such as co-occurrence network analysis—can rapidly identify hotspots and interests of investigators in HPV pathogenicity from large volumes of literature. However, these targets may lack experimental validation (e.g., gain- or loss-of-function studies) or may be biologically undruggable. Therefore, bibliometric findings require careful filtering and critical evaluation to avoid misleading conclusions. A comprehensive understanding of the field benefits from combining bibliometric analysis of historical trends with systematic reviews of recent mechanistic and clinical evidence. Both approaches are essential and complementary in guiding future research.

In summary, this study not only charts the historical development of the field but also provides a strategic foundation to guide future endeavors. By leveraging mechanistic insights and embracing interdisciplinary collaboration, the research community can accelerate the development of effective antiviral therapies to alleviate the global burden of HPV-associated cancers.

## Figures and Tables

**Table 1:** Basic information of the publications.

Articles	Proceeding	Early Access	Others	Authors	Institutions	Journals	Subject
2114	73	10	1	10,885	2920	726	88

**Table 2:** Top 10 journals from 1985 to 2005.

Journal	Counts	5-Year IF	Category	Quartile	Publisher
Journal of Virology	81	3.5	Virology	Q2	AMER SOC MICROBIOLOGY
Plos one	55	3.2	Multidisciplinary Science	Q2	PUBLIC LIBARARY SCIENCE
International Journal of Cancer	45	5.9	Oncology	Q1	WILLEY
Vaccine	44	3.5	Immunology; Medicine, Research, experimental	Q2	ELSVIER
Antiviral Research	32	4.3	Pharmacology & Pharmacy, Virology	Q1	ELSVIER
Gynecologic oncology	30	4.4	Obstetrics & Gynecology	Q1	ELSEVIER
Journal of Medical Virology	26	4.7	Virology	Q1	WILLEY
Virology	26	2.5	Virology	Q3	ELSEVIER
Scientific Reports	23	4.3	MULTIDISCIPLINARY SCIENCE	Q1	NATURE PORTFOLIO
Frontiers in Immunology	22	6.8	IMMUNOLOGY	Q1	FRONTIERS MEIDA SA

**Table 3:** List of top 25 most prolific authors.

Author	Publications	Country	Affiliation
S.H. van der Burg	15	Netherland	Leiden University
Stephen K. Tyring	14	USA	University of Texas Medical Branch
Azam Bolhassani	11	Iran	Pasteur Institute of Iran
Arany Istvan	11	USA	University of Texas Medical Branch
Clint T. Allen	10	USA	National Institutes of Health
Iain M. Morgan	9	USA	Virginia Commonwealth University
Cornelis (Kees) J.M. Melief	8	Netherland	Leiden University
Donalisio Manuela	8	Italy	University of Turin
Claire D. James	8	USA	Virginia Commonwealth University
Edith M. G. van Esch	7	Netherland	Catharina Hospital Eindhoven
Xu Wang	7	USA	Emory University School of Medicine
David Lembo	7	Italy	University of Turin
Allan L. Abramson	7	USA	Long Island Jewish Medical Center
Erik De Clercq	7	Belgium	Universiteit Leuven
Hung, Chien-Fu	7	USA	Johns Hopkins University
Mariëtte I.E. van Poelgeest	6	Netherland	Leiden University
Andrea Civra	6	Italy	San Luigi Gonzaga Hospital
Graciela Andrei	6	Belgium	Katholieke Universiteit
Jeffrey Schlom	6	USA	National Institutes of Health
Cornelia L. Trimble,	6	USA	Johns Hopkins University
Vincent R. Bonagura	6	USA	Feinstein Institute for Medical Research
John T. Schiller	6	USA	National Institutes of Health
Haim Abramovici	6	Israel	Carmel Medical Center
Robert Snoeck	6	Belgium	Katholieke Universiteit
Jocab Bornstein	6	Israel	Bar Ilan University

## Data Availability

The original contributions presented in this study are included in the article/Supplementary Materials. Further inquiries can be directed to the corresponding authors.

## Supplementary Materials

The following supporting information can be downloaded at: https://www.mdpi.com/article/10.3390/pathogens15030265/s1.

## Abbreviations

The following abbreviations are used in this manuscript:

HPV	Human papillomavirus
USD	United States Dollar
pRB	Retinoblastoma protein
IFN	Interferon
cGAS-STING	Cyclic GMP-AMP synthase–stimulator of interferon genes
WHO	World Health Organization
WoSCC	Web of Science Core Collection
COVID-19	Coronavirus disease 2019
NIH	National Institutes of Health
NCI	National Cancer Institute
IRF-3	Interferon Regulatory Factor 3
IRF-1	Interferon Regulatory Factor 1
Tyk2	Tyrosine kinase 2
STAT-1	Signal Transducer and Activator of Transcription 1
Th2	Helper T cell 2
TLR7	Toll-like receptor 7
Th1	Helper T cell 1
JAK	Janus kinases
NF-κB	Nuclear factor kappa-light-chain-enhancer of activated B cells
PCR	Polymerase chain reaction
CRISPR	Clustered Regularly Interspaced Short Palindromic Repeats
VLPs	Virus-like particles
